# The impact of comorbid headache on surgical outcomes in drug-resistant focal epilepsy

**DOI:** 10.3389/fneur.2026.1964217

**Published:** 2026-10-23

**Authors:** Wenchao Cheng, Aochen Shi, Maiqi Du, Yunqing Ma, Yuhan Wang, Hongliang Jiao, Jiefeng Geng, Yajun Lian, Yi Tian, Xiaolei Lian

**Affiliations:** 1 Department of Neurology, The First Affiliated Hospital of Zhengzhou University, Zhengzhou, Henan, China; 2 Department of Neurology, The Second Affiliated Hospital of Zhengzhou University, Zhengzhou, Henan, China; 3 Department of Neurosurgery, The First Affiliated Hospital of Zhengzhou University, Zhengzhou, Henan, China

**Keywords:** comorbid headache, drug-resistant focal epilepsy, epilepsy surgery, surgical outcomes, unilateral headache

## Abstract

**Background:**

Headache is a common comorbidity of epilepsy and is closely associated with the poor prognosis of epilepsy. Resection surgery of the epileptogenic focus is an effective treatment method for drug-resistant focal epilepsy (DRFE). However, the relationship between headache and surgical outcomes remains unclear. This study aimed to investigate the impact of comorbid headache and its characteristics on short-term surgical outcomes in DRFE.

**Methods:**

DRFE patients undergoing resection surgery were consecutively enrolled and followed up for at least one year after surgery. Seizure-free rate within 1 year after surgery was compared between the headache and non-headache groups. Subgroup analysis was performed within the headache group to compare headache characteristics between patients who were seizure-free and those who experienced seizures within 1 year after surgery.

**Results:**

A total of 103 DRFE patients were enrolled, among whom 46 (44.7%) had comorbid headache. Of the 46 patients, 23 experienced unilateral headache, and in 22 of these 23 patients (22/23, 95.7%), the headache side was ipsilateral to the epileptogenic focus. The difference in the seizure-free rate within 1 year after surgery between the headache and non-headache groups was not statistically significant (78.3% vs. 73.7%, *p* = 0.590). Among DRFE patients with comorbid headache, compared with the seizure within 1 year after surgery group, unilateral headache was significantly more frequent (58.3% vs. 20.0%, *p* = 0.032), and the mean preoperative visual analogue scale (VAS) score was significantly lower (5.50 ± 2.171 vs. 7.60 ± 1.578, *p* = 0.007) in the seizure-free within 1 year after surgery group. Multivariate binary logistic regression analysis demonstrated that unilateral headache was independently associated with higher odds of seizure-free within 1 year after surgery (OR = 1.854, 95% CI 1.112–3.092, *p* = 0.018), whereas the preoperative VAS score was independently associated with lower odds of seizure-free within 1 year after surgery (OR = 0.136, 95% CI 0.020–0.923, *p* = 0.041).

**Conclusion:**

Comorbid headache does not significantly affect short-term surgical outcomes in DRFE patients. However, for DRFE patients with comorbid headache, unilateral headache or a lower preoperative VAS score may serve as potential clinical indicators for favorable short-term surgical outcomes.

## 1 Introduction

Epilepsy is a chronic brain disorder caused by abnormally excessive and synchronous neuronal discharge, characterized by a persistent predisposition to generate epileptic seizures. Currently, there are approximately 70 million people with epilepsy worldwide, with over 10 million in China alone (1, 2). According to statistics, approximately 50% of adults with epilepsy suffer from one or more comorbid conditions, with headache (especially migraine) being one of the most common (3–5). The risk of headache is significantly higher in people with epilepsy than in the general population. A recent meta-analysis showed that the prevalence of headache in epilepsy patients is approximately 49% (6). Another meta-analysis indicated that the lifetime prevalence of migraine is 52% higher in people with epilepsy compared to those without, and conversely, the lifetime prevalence of epilepsy is 79% higher in individuals with migraine compared to those without (7). According to newly released data from the Global Burden of Disease (GBD), migraine and epilepsy rank third and seventh among neurological disorders, respectively. However, in the population aged 5 to 19 years, migraine has jumped to the first place, while epilepsy has risen to the third place (8). Thus, epilepsy comorbid with headache imposes not only a heavy psychological burden on patients but also significant economic pressure on their families and society.

Despite receiving adequate and appropriate courses of anti-seizure medications (ASMs), approximately one-third of patients still fail to achieve complete seizure control and subsequently progress to drug-resistant epilepsy, with drug-resistant focal epilepsy (DRFE) being the most common type (9, 10). DRFE severely impacts epilepsy patients on physiological, psychological, and social levels. First, patients with DRFE often experience frequent seizures or even status epilepticus, leading to significantly reduced cognitive function, quality of life, and increased mortality risk (11). Second, DRFE patients frequently have comorbid mental disorders such as anxiety and depression, which can reduce treatment adherence and potentially worsen the condition (12, 13). Furthermore, DRFE often leads to interruption of education and employment, restricted daily activities, and social phobia, severely hindering personal development. Resection surgery is an effective treatment for DRFE. Especially when the epileptogenic focus is well-localized, resection surgery can significantly increase the seizure-free rate, improve quality of life, and reduce mortality risk in DRFE patients (10). However, the success rate of surgery for drug-resistant epilepsy remains variable (14).

There are extensive pathophysiological links between headache and epilepsy, with shared mechanisms including cortical spreading depression (CSD), neurotransmitter alterations, genetic susceptibility genes (e.g., *CACNA1A, ATP1A2, SCN1A*), and common triggering factors such as stress, fatigue, and alcohol consumption (5). Among the different headache types, migraine in particular has been shown to significantly increase seizure frequency, reduce responsiveness to ASMs, and be associated with a higher proportion of drug-resistant epilepsy, thereby adversely affecting the overall prognosis of epilepsy patients (15–18). Although DRFE patients have a high comorbidity rate of headache, previous research has mostly focused on the impact of headache on the prognosis of non-surgical epilepsy patients, while the relationship between headache and surgical outcomes in DRFE patients remains unclear. By comparing the postoperative seizure recovery status of DRFE patients with and without comorbid headache, this study aims to investigate the impact of headache and its clinical characteristics on short-term surgical outcomes in DRFE patients, expecting to provide a reference for clinical management and preoperative evaluation of DRFE patients with comorbid headache.

## 2 Methods

### 2.1 Study participants

This study consecutively enrolled patients with DRFE who underwent resection surgery of the epileptogenic focus at the First Affiliated Hospital of Zhengzhou University from April 2020 to December 2023. Preoperative clinical data were collected using face-to-face structured questionnaire surveys. Postoperative follow-up was conducted for at least one year via outpatient visits, hospitalization, or telephone interviews. All patients or their legal guardians signed the informed consent forms. This study was approved by the Ethics Committee of the First Affiliated Hospital of Zhengzhou University (Ethics Approval Number: 2024-KY-1585).

Inclusion criteria: (a) Met the diagnostic criteria for drug-resistant epilepsy proposed by the International League Against Epilepsy (ILAE) in 2010, i.e., failure to achieve sustained seizure freedom for 12 months or failure to achieve a seizure-free period equivalent to three times the longest pre-treatment inter-seizure interval after adequate trials of at least two appropriately selected and correctly used ASMs (19); (b) Complete clinical data; (c) Definite diagnosis of DRFE assessed by a multidisciplinary epilepsy specialist team, meeting the indications for resection surgery of the epileptogenic focus and receiving surgical treatment.

Exclusion criteria: (a) Patients with unclear epileptogenic focus or deemed unsuitable for resection surgery after comprehensive clinical evaluation; (b) Patients with missing essential clinical data; (c) Patients unable to recall or clearly describe their headache status; (d) Patients unable to complete follow-up due to reasons such as intellectual disability and mental abnormality. (e) Patients who refused to cooperate with follow-up after surgery. No patient was excluded on the basis of headache type or subtype. All headache types or subtypes were eligible for inclusion, provided the patient could reliably describe the headache characteristics and complete follow-up.

### 2.2 Data collection

All patients were evaluated by a multidisciplinary epilepsy specialist team. Based on comprehensive assessment of seizure semiology, long-term video-electroencephalography (VEEG), at least three habitual seizure manifestations and ictal EEG, high-resolution brain magnetic resonance imaging (MRI), positron emission tomography-computed tomography (PET-CT), and imaging post-processing fusion, the epileptogenic focus was determined. When necessary, stereo electroencephalography (SEEG) was used intraoperatively to further localize the epileptogenic focus. Based on intraoperative electrocorticography (ECoG) and brain mapping results, essential functional cortex areas requiring preservation were identified, and maximal resection of the epileptogenic cortex was performed while protecting functional cortex as much as possible.

Baseline data and clinical characteristics were collected for all patients, including age, sex, epilepsy duration, age at epilepsy onset, brain MRI findings, focus location, seizure type, seizure frequency, and family history of epilepsy and headache. For patients with comorbid headache, additional information was collected regarding the temporal relationship between headache and seizures, as well as the headache’s location, nature, intensity, associated symptoms, and frequency. The visual analogue scale (VAS) was used to quantify headache intensity.

### 2.3 Classification of headache

Headache was classified into migraine, tension-type headache, and unclassified headache according to the diagnostic criteria of the International Classification of Headache Disorders-3 (ICHD-3) issued by the Headache Classification Committee of the International Headache Society. Additionally, based on the temporal relationship with seizures, headache was classified as peri-ictal headache or interictal headache. Peri-ictal headache included preictal headache, ictal headache, and postictal headache. Preictal headache was defined as headache starting within 24 h before a seizure and continuing into the seizure. Ictal headache was defined as headache occurring simultaneously with the seizure, with significant relief or disappearance of headache after seizure cessation. Postictal headache was defined as headache starting within 3 h after a seizure and resolving spontaneously within 72 h after seizure cessation. Interictal headache was defined as headache and seizure occurring independently, with headache onset not earlier than 3 h after a seizure, or headache that did not develop into a seizure. For the subgroup analysis, each temporal phase was considered separately. Single-phase headache was defined as headache occurring in only one temporal phase (preictal, ictal, postictal, or interictal). Multi-phase headache was defined as headache occurring in two or more of these phases.

### 2.4 Statistical analysis

SPSS 27.0 statistical software was used for data processing. For quantitative data, the Shapiro–Wilk test was used for normality testing. Data following a normal distribution were described as mean ± standard deviation (mean ± SD), and intergroup comparisons were performed using the Student’s t test. Data not following a normal distribution were described as median (interquartile range) [M (P25, P75)], and intergroup comparisons were performed using Mann–Whitney U test. Qualitative data were described as number of cases (percentage) [*n* (%)], and intergroup comparisons were performed using the chi-square test or Fisher’s exact test. In patients with comorbid headache, univariate analysis was used to assess the impact of each headache characteristic on surgical outcomes, and statistically significant factors were included in a binary logistic regression model. A *p*-value < 0.05 was considered statistically significant.

## 3 Results

### 3.1 General data

During the study period (April 2020 to December 2023), 132 patients with DRFE were initially screened. Of these, 8 patients had an unclear epileptogenic focus or were deemed unsuitable for resection surgery after multidisciplinary evaluation, 6 patients had missing essential clinical data, 5 patients were unable to recall or clearly describe their headache status, 4 patients were unable to complete follow-up due to intellectual disability or mental abnormality, and 6 patients refused to cooperate with postoperative follow-up. Finally, 103 patients were enrolled and included in the analysis. Among them, 76 had temporal lobe epilepsy, 17 had frontal lobe epilepsy, 6 had parietal lobe epilepsy, and 4 had occipital lobe epilepsy. There were 57 males (55.3%) and 46 females (44.7%). Median age was 24 years (range: 4–55 years), median epilepsy duration was 9 years (range: 1–40 years), and median age at epilepsy onset was 11 years (range: 9 months–51 years).

### 3.2 Distribution of headache types

Among the 103 DRFE patients, 46 patients (46/103, 44.7%) had comorbid headache, including 22 with migraine (22/103, 21.4%), 13 with tension-type headache (13/103, 12.6%), 9 with unclassified headache (9/103, 8.7%), and 2 patients (2/103, 1.9%) had both migraine and tension-type headache.

According to the temporal relationship between headache and seizures, among the 46 patients with comorbid headache, 14 had interictal headache (14/103, 13.6%), and 41 had peri-ictal headache (41/103, 39.8%). Among these 41 patients with peri-ictal headache, there were 4 with preictal headache (4/103, 3.9%), 38 with postictal headache (38/103, 36.9%), and 1 with ictal headache (1/103, 1.0%). Ten patients (10/46, 21.7%) had two or more types of headache, with the coexistence of postictal and interictal headache being the most common (8/10, 80.0%) (Figure 1).

**Figure 1:**
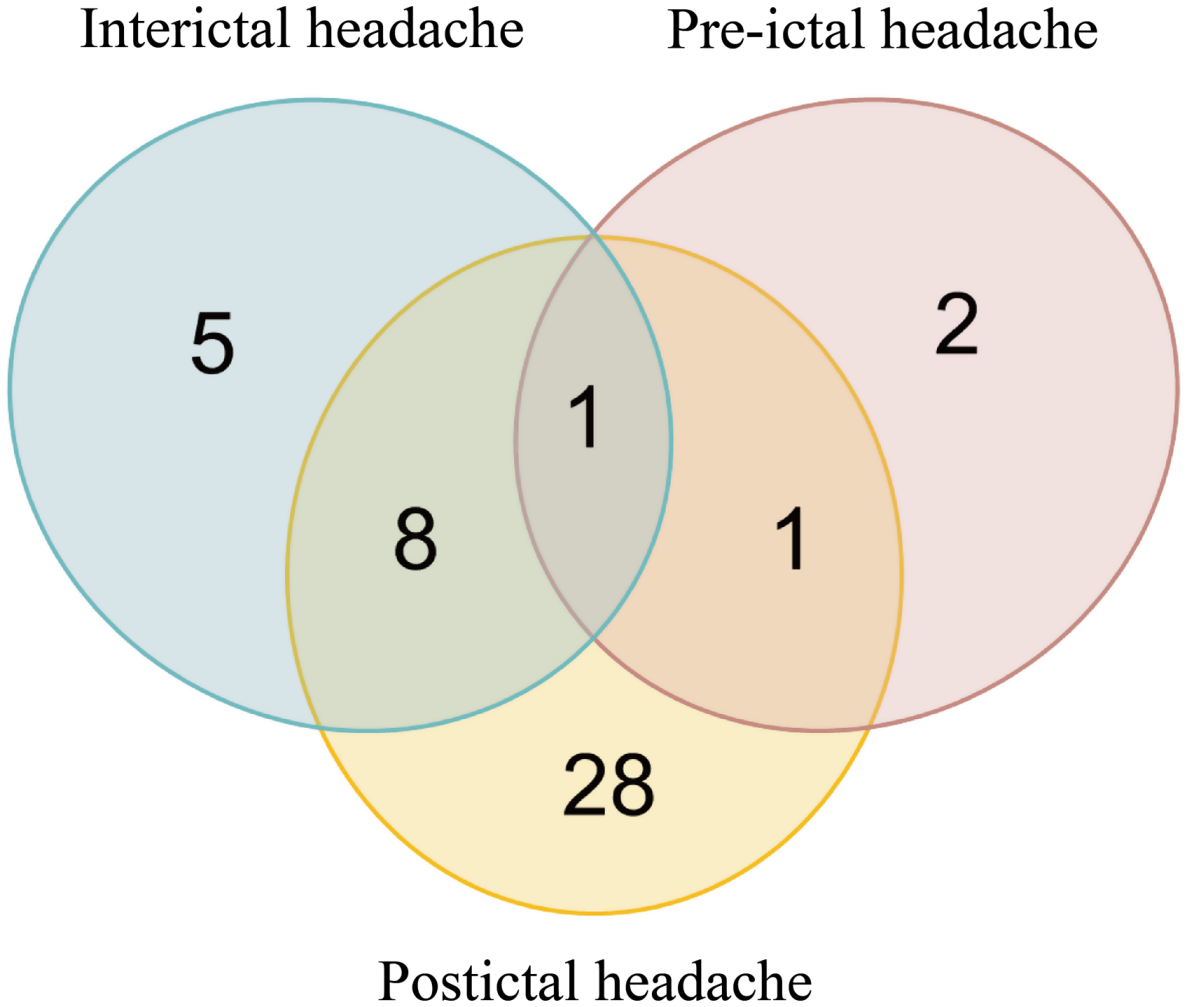
Distribution of various types of headaches.

Notably, among the 46 patients with comorbid headache, 23 had unilateral headache, and in 22 of these patients (22/23, 95.7%), the side of headache was ipsilateral to the epileptogenic focus.

### 3.3 Intergroup comparison of clinical characteristics

#### 3.3.1 Comparison of clinical characteristics between headache and non-headache groups

Patients were divided into a headache group (46 cases) and a non-headache group (57 cases) based on the presence of preoperative comorbid headache. No statistically significant differences were found between the two groups in terms of age, age at epilepsy onset, epilepsy duration, seizure type, focus location, brain MRI results, or family history of epilepsy (*p* > 0.05). Compared with the non-headache group, the headache group had a significantly higher proportion of female patients (56.5% vs. 35.1%, *p* = 0.030), and a significantly higher proportion of patients with a family history of headache (15.2% vs. 0.0%, *p* = 0.003). These differences were statistically significant. Furthermore, there was also a statistically significant difference in preoperative seizure frequency between the two groups (*p* = 0.027), with the headache group having a significantly higher proportion of patients with a seizure frequency of ≥ 1 time/week than the non-headache group (50.0% vs. 24.6%) (Table 1).

**Table 1:** Comparison of clinical characteristics between the headache group and the non-headache group.

Clinical characteristics	Headache (*n* = 46)	Non-headache (*n* = 57)	*p* value
Female [*n* (%)]	26 (56.5%)	20 (35.1%)	**0.030**
Age [years, M (Q1, Q3)]	24.5 (14, 35)	23 (14.5, 34)	0.950
Age at epilepsy onset [years, M (Q1, Q3)]	10 (5, 21)	12 (5, 20)	0.581
Epilepsy duration [years, M (Q1, Q3)]	9 (4, 20)	8 (4, 18)	0.695
Seizure type [*n* (%)]			0.538
Focal seizure	11 (23.9%)	19 (33.3%)	
Generalized seizure	7 (15.2%)	9 (15.8%)	
Both	28 (60.9%)	29 (50.9%)	
Preoperative seizure frequency [*n* (%)]			**0.027**
< 1 time/month	12 (26.1%)	24 (42.1%)	
≥ 1 time/month, < 1 time/week	11 (23.9%)	19 (33.3%)	
≥ 1 time/week	23 (50.0%)	14 (24.6%)	
Focus location [*n* (%)]			0.568
Frontal lobe	6 (13.0%)	11 (19.3%)	
Temporal lobe	34 (73.9%)	42 (73.7%)	
Parietal lobe	3 (6.5%)	3 (5.3%)	
Occipital lobe	3 (6.5%)	1 (1.8%)	
Negative brain MRI [*n* (%)]	13 (28.3%)	10 (17.5%)	0.194
Family history of epilepsy [*n* (%)]	4 (8.7%)	4 (7.0%)	> 0.999
Family history of headache [*n* (%)]	7 (15.2%)	0 (0.0%)	**0.003**

Bold values indicate statistical significance (*p* < 0.05).

#### 3.3.2 Comparison of clinical characteristics between single-phase and multi-phase headache groups

To determine whether the temporal pattern of headache, beyond its mere presence, is associated with distinct clinical characteristics in DRFE, following the operational definitions in Methods 2.3, the 46 patients with comorbid headache were divided into a single-phase headache group (36 cases) and a multi-phase headache group (10 cases). No statistically significant differences were found between the two groups in terms of sex, age, age at epilepsy onset, epilepsy duration, seizure type, seizure frequency, focus location, brain MRI results, or family history of epilepsy (*p* > 0.05). Compared with the single-phase headache group, the multi-phase headache group had a significantly higher proportion of patients with a family history of headache (40.0% vs. 8.3%, *p* = 0.031; Table 2), suggesting that multi-phase headache may reflect a stronger familial predisposition and may be useful for preoperative comorbidity assessment.

**Table 2:** Comparison of clinical characteristics between the single-phase headache group and the multi-phase headache group.

Clinical characteristics	Single-phase headache (*n* = 36)	Multi-phase headache (*n* = 10)	*p* value
Female [*n* (%)]	20 (55.6%)	6 (60.0%)	> 0.999
Age [years, M (Q1, Q3)]	27.5 (14, 37)	19.5 (12.75, 26.25)	0.116
Age at epilepsy onset [years, M (Q1, Q3)]	11 (6, 22)	5 (3, 16.5)	0.088
Epilepsy duration [years, M (Q1, Q3)]	9 (4.25, 20.75)	9 (4, 18)	> 0.999
Seizure type [*n* (%)]			0.143
Focal seizure	10 (27.8%)	1 (10.0%)	
Generalized seizure	7 (19.4%)	0 (0.0%)	
Both	19 (52.8%)	9 (90.0%)	
Preoperative seizure frequency [*n* (%)]			0.522
< 1 time/month	11 (30.6%)	1 (10.0%)	
≥ 1 time/month, < 1 time/week	8 (22.2%)	3 (30.0%)	
≥ 1 time/week	17 (47.2%)	6 (60.0%)	
Focus location [*n* (%)]			0.106
Frontal lobe	3 (8.3%)	3 (30.0%)	
Temporal lobe	29 (80.6%)	5 (50.0%)	
Parietal lobe	2 (5.6%)	1 (10.0%)	
Occipital lobe	2 (5.6%)	1 (10.0%)	
Negative brain MRI [*n* (%)]	10 (27.8%)	3 (30.0%)	> 0.999
Family history of epilepsy [*n* (%)]	3 (8.3%)	1 (10.0%)	> 0.999
Family history of headache [*n* (%)]	3 (8.3%)	4 (40.0%)	**0.031**

Bold values indicate statistical significance (*p* < 0.05).

### 3.4 Surgical outcomes

#### 3.4.1 Impact of headache and its subtypes on the surgical outcomes of epilepsy

Among the 103 DRFE patients, 78 (78/103, 75.7%) were seizure-free within 1 year after surgery, while 25 (25/103, 24.3%) still had seizures. The seizure-free rates within 1 year after surgery were 78.3% (36/46) in the headache group and 73.7% (42/57) in the non-headache group. The rates were 80.5% (33/41) in the peri-ictal headache group and 78.6% (11/14) in the interictal headache group. The rates were 90.0% (9/10) in the multi-phase headache group and 75.0% (27/36) in the single-phase headache group. The rates were 86.4% (19/22) in the migraine group, 92.3% (12/13) in the tension-type headache group, and 77.8% (7/9) in the unclassified headache group. None of these differences were statistically significant (*p* > 0.05) (Table 3).

**Table 3:** The impact of headache and its subtypes on the surgical outcomes of epilepsy.

Headache subtypes	Seizure-free within 1 year after surgery	Seizure within 1 year after surgery	*p* value
Non-headache	42	15	0.590
Headache	36	10	
Peri-ictal headache	33	8	0.877
Interictal headache	11	3	
Multi-phase headache	9	1	0.309
Single-phase headache	27	9	
Migraine	19	3	0.621
Tension-type headache	12	1	
Unclassified headache	7	2	

#### 3.4.2 Impact of headache characteristics on the surgical outcomes of epilepsy in patients with comorbid headache

Next, we further analyzed the impact of headache characteristics on the surgical outcomes of epilepsy in 46 patients with comorbid headache. Univariate analysis results (Table 4) showed that 58.3% (21/36) of patients had unilateral headache in the seizure-free within 1 year after surgery group, whereas only 20.0% (2/10) had unilateral headache in the seizure within 1 year after surgery group. The difference between the two groups was statistically significant (*p* = 0.032). The preoperative VAS score in the seizure-free within 1 year after surgery group (5.50 ± 2.171) was significantly lower than that in the seizure within 1 year after surgery group (7.60 ± 1.578), and this difference was also statistically significant (*p* = 0.007). No statistically significant differences were found between the two groups in terms of headache nature, associated symptoms, or family history of headache (*p* > 0.05).

**Table 4:** Univariate analysis of the impact of headache characteristics on the surgical outcomes of epilepsy in patients with comorbid headache.

Headache characteristics	Seizure-free within 1 year after surgery (*n* = 36)	Seizure within 1 year after surgery (*n* = 10)	*p* value
Unilateral headache [*n* (%)]	21 (58.3%)	2 (20.0%)	**0.032**
Headache nature [*n* (%)]			0.555
Distending	19 (52.8%)	4 (40.0%)	
Throbbing	3 (8.3%)	0 (0.00%)	
Tightening	6 (16.7%)	2 (20.0%)	
Others	8 (22.2%)	4 (40.0%)	
VAS score (mean ± SD)	5.50 ± 2.171	7.60 ± 1.578	**0.007**
Nausea and/or vomiting [*n* (%)]	17 (47.2%)	5 (50.0%)	> 0.999
Photophobia and/or phonophobia [*n* (%)]	13 (36.1%)	3 (30.0%)	> 0.999
Family history of headache [*n* (%)]	5 (13.9%)	1 (10.0%)	> 0.999

Bold values indicate statistical significance (*p* < 0.05).

Variables with statistical significance (unilateral headache and VAS score) in the univariate analysis were included in a multivariate binary logistic regression analysis. The results showed that unilateral headache was independently associated with higher odds of seizure-free within 1 year after surgery (OR = 1.854, 95% CI 1.112–3.092, *p* = 0.018), whereas the preoperative headache VAS score was independently associated with lower odds of seizure-free within 1 year after surgery (OR = 0.136, 95% CI 0.020–0.923, *p* = 0.041) (Table 5).

**Table 5:** Multivariate analysis of the impact of headache characteristics on the surgical outcomes of epilepsy in patients with comorbid headache.

Headache characteristics	OR	95% CI	*p* value
Unilateral headache	1.854	1.112–3.092	**0.018**
VAS score	0.136	0.020–0.923	**0.041**

Bold values indicate statistical significance (*p* < 0.05).

#### 3.4.3 Surgical outcomes of headache

Among the 41 patients with peri-ictal headache, 33 were seizure-free within 1 year after surgery, and their peri-ictal headache consequently resolved. The remaining 8 patients still had seizures within 1 year after surgery; 7 of them also experienced peri-ictal headache, and in 1 patient, the postoperative seizure type changed from generalized tonic–clonic seizure to focal seizure, with no recurrence of peri-ictal headache.

Among the 14 patients with interictal headache, 11 were seizure-free within 1 year after surgery. The surgical outcomes of their headache were as follows: In 5 patients, age at epilepsy onset was earlier than age at interictal headache onset, and interictal headache resolved after surgery. In 4 patients, age at interictal headache onset was earlier than age at epilepsy onset, and headache showed no significant improvement after surgery. In the other 2 patients, the ages at onset for both epilepsy and interictal headache were very young, making it difficult to determine the temporal sequence; in both these patients, headache resolved after surgery. Among the remaining 3 patients who still had seizures within 1 year after surgery, 2 showed no significant improvement in interictal headache after surgery, and 1 had resolution of interictal headache after surgery.

## 4 Discussion

This study aimed to investigate the impact of comorbid headache and its clinical characteristics on short-term surgical outcomes in DRFE patients by comparing the seizure-free rates within 1 year after surgery between patients with and without comorbid headache, and by conducting subgroup analysis on those with comorbid headache. The results showed no significant difference in the seizure-free rates within 1 year after surgery between the headache group and the non-headache group. However, subgroup analysis further revealed that among DRFE patients with comorbid headache, unilateral headache was independently associated with higher odds of seizure-free within 1 year after surgery, whereas a higher preoperative VAS score was independently associated with lower odds of seizure-free within 1 year after surgery.

A previous retrospective study systematically analyzing literature on epilepsy comorbid with headache, published between 2014 and 2019, indicated that postictal headache is the most common type of peri-ictal headache, while preictal headache is seen in only 1–10% of epilepsy patients, and ictal headache is extremely rare (5). Another study, involving EEG confirmation in 831 epilepsy patients, identified only 6 cases (0.7%) of ictal headache (20). Our results are similar: the proportions of preictal, postictal, and interictal headache were 3.9%, 36.9%, and 13.6%, respectively, and we identified only 1 case of ictal headache. Furthermore, 21.7% (10/46) of patients with comorbid headache were not limited to a single type but experienced headaches in different temporal phases, with the combination of postictal and interictal headache being the most common (80%, 8/10). Patients with a family history of headache were more likely to experience multi-phase headache.

Cortical spreading depression (CSD) is an important mechanism underlying the comorbidity of headache and epilepsy (20–22). It is characterized by a brief period of cortical excitation followed by prolonged cortical activity suppression (23). CSD can activate the trigeminovascular system (TVS), triggering a cascade release of numerous inflammatory molecules and neurotransmitters, thereby inducing the pain of a migraine attack (24). The threshold for the abnormal neuronal electrical activity required for epileptic seizures is higher than the threshold for inducing CSD. Therefore, seizures directly triggered by migraine are very rare, whereas comorbid migraine is more common in epilepsy patients. Especially in DRFE patients, recurrent seizures may facilitate the occurrence of CSD, further increasing the prevalence of migraine. This study also confirmed that the total comorbidity rate of headache in DRFE patients undergoing surgery was 44.7%, with migraine comorbidity at 21.4%, significantly higher than tension-type headache (12.6%) and unclassified headache (8.7%).

A previous study has shown that among patients with temporal lobe epilepsy or extratemporal lobe epilepsy who have comorbid unilateral peri-ictal headache, 93.75 and 70%, respectively, have headache ipsilateral to the epileptogenic focus; among patients with unilateral interictal headache, 90 and 83.3%, respectively, have headache ipsilateral to the epileptogenic focus (25). Bernasconi et al. also found that in temporal lobe epilepsy patients with comorbid unilateral peri-ictal headache, 90% had headache ipsilateral to the epileptogenic focus (26). Our results are consistent: among the 46 patients with comorbid headache, 23 presented with unilateral headache, and in 22 of these (95.7%), the headache side was ipsilateral to the epileptogenic focus. This evidence further indicates a strong spatial correlation between unilateral headache and the epileptogenic focus, suggesting that unilateral headache has lateralizing value for the epileptogenic focus.

By comparing the clinical characteristics of the headache and non-headache groups, this study found that DRFE patients who are female, have a family history of headache, or have a seizure frequency of ≥ 1 time/week are more likely to have comorbid headache. Specifically, the higher proportion of female patients in the headache group is consistent with the generally higher susceptibility to headache in women. Previous studies have suggested that hormonal fluctuations, particularly during the postpartum period and late luteal phase, may contribute to migraine risk through effects on serotonergic and GABAergic activity (27). Additionally, DRFE patients with a family history of headache had a higher rate of comorbid headache, suggesting a significant role for genetic factors. Both epilepsy and migraine have high hereditary susceptibility, especially idiopathic epilepsy and migraine with aura, which may share common causative genes (17). A previous large cross-sectional study also indicated that females and patients with a family history of migraine or epilepsy are more prone to peri-ictal headache (18). However, this study did not find a significant association between family history of epilepsy and comorbid headache. Gameleira et al. found that patients with frequent seizures were more likely to have comorbid headache (28), consistent with our findings. In contrast, although Wang et al. did not observe a significant association between headache and seizure frequency, their study confirmed a high prevalence of postictal headache, suggesting that headache is often triggered by epileptic seizures (29).

Velioğlu et al. found that compared to epilepsy patients without migraine, those with comorbid migraine had poorer early treatment response and lower seizure-free rates during 10-year follow-up, suggesting a negative impact of migraine on epilepsy prognosis (30). However, research on the impact of comorbid headache on the surgical outcomes of epilepsy patients remains limited. This study compared the short-term surgical outcomes of DRFE patients in the headache and non-headache groups, revealing no significant difference in the seizure-free rate within 1 year after surgery, which might be related to the relatively short follow-up period. To our knowledge, this study is the first to investigate the impact of preoperative headache characteristics on the surgical outcomes of DRFE patients with comorbid headache, finding that in DRFE patients with comorbid headache, unilateral headache was independently associated with higher odds of seizure-free within 1 year after surgery, whereas a higher preoperative VAS score was independently associated with lower odds of seizure-free within 1 year after surgery. A previous study has shown that chronic abnormal electrical activity in the brain can lead to an imbalance in neuronal membrane excitability, increasing susceptibility to headache (31). Currently, CSD and TVS activation are recognized as the two core pathophysiological mechanisms underlying the comorbidity of epilepsy and headache. The abnormal discharges generated by the localized epileptogenic focus may trigger CSD, and CSD can further activate TVS, resulting in unilateral headache (25, 27, 32–34). CSD can sometimes spread from one cerebral hemisphere to the other (35, 36), which explains the mechanism of bilateral headache. It has also been reported that the local vasodilation and reactive hyperemia at the site of localized epileptogenic focus may lead to the activation of trigeminal sensory system and therefore cause unilateral headache (25). Additionally, headache can be directly evoked by the electrical stimulation of the anterior cingulate cortex, the entorhinal cortex, the anterior and posterior insula, suggesting that unilateral headache is closely related to abnormal discharges generated by the localized epileptogenic focus (37). Moreover, as mentioned before, previous studies and our own research have both shown that unilateral headache has lateralizing value for the epileptogenic focus (17, 18). Therefore, unilateral headache might reflect more localized epileptogenic networks compared to bilateral headache, thus indicating a better surgical outcome. Furthermore, the headache VAS score can reflect, to some extent, the intensity of TVS activation by CSD. CSD can induce cerebral ischemia, hypoxia, and energy metabolism disorders, while TVS activation causes the release of inflammatory mediators, both of which can lead to brain cell damage (38, 39). This could explain the poorer surgical outcomes in patients with high VAS scores.

While achieving seizure freedom within 1 year after surgery is a significant milestone, it does not guarantee sustained long-term control. Long-term follow-up data consistently show that the proportion of patients remaining completely seizure-free declines over time (40, 41). Despite the risk of late recurrence, maintaining short-term seizure freedom is strongly associated with long-term seizure freedom. In pediatric epilepsy surgery, the chance of long-term seizure freedom increases to 87% if no seizure occurs within 1 year after surgery (42). Seizure recurrence beyond the first postoperative year exhibits significant variability, with both early and late patterns observed. Early recurrences are more common, with 92% of recurrences occurring within the first 3 years postoperatively in one anterior temporal lobectomy cohort. However, late recurrences can occur decades after surgery, with reports of first recurrences up to 23 years postoperatively (40). Late recurrences tend to follow a milder course compared to early recurrences, and patients are often able to regain remission with medical management (43). Moreover, the annual probability of seizure recurrence varies over time. Among patients who are seizure-free at the end of the first postoperative year, the annual probability of seizure recurrence is 5.6% between years 1 and 5, and decreases to 4.2% beyond year 5 (44). This temporal pattern underscores the importance of ongoing monitoring and follow-up, even for patients who achieve early success, to promptly identify and manage late recurrences.

During follow-up, we observed that some patients did not understand the purpose of inquiring about headaches, believing that headache is unrelated to epilepsy. In China, most headache patients do not seek professional medical help and tend to take over-the-counter analgesic medications to relieve symptoms. This situation highlights that clinicians should pay attention to the headache problems of epilepsy patients (29). A detailed inquiry into headache characteristics before epilepsy surgery has important guiding value for preoperative evaluation. Although the comorbidity of epilepsy (especially drug-resistant epilepsy) with headache is very common, and sometimes the headache itself can be more disabling than the seizures, research in this area remains relatively limited. Therefore, conducting in-depth research on epilepsy comorbid with headache and strengthening comorbidity management in clinical practice is of great practical significance.

This study has several limitations: (a) As a single-center study with a limited sample size, selection bias may exist. The generalizability of the findings needs to be verified by multi-center, large-sample studies. (b) The postoperative follow-up period was only one year, representing short-term outcome assessment. The impact of comorbid headache on long-term surgical outcomes could not be observed. (c) DRFE patients typically need to take multiple ASMs preoperatively, with individual differences in medication regimens, doses, and durations. This study did not systematically control for the potential impact of ASM types and exposure duration on comorbid headache and surgical outcomes. (d) although preoperative headache data were collected face-to-face using a structured questionnaire and classified according to ICHD-3, headache diagnosis and temporal relationship to seizures inevitably depend on patient recall. Recall bias may therefore have influenced the classification of headache type (migraine, tension-type, or unclassified), and temporal pattern (preictal, ictal, postictal, or interictal).

## 5 Conclusion

This study did not find a significant impact of comorbid headache on the seizure-free rate within 1 year after surgery in DRFE patients. However, among DRFE patients with comorbid headache, preoperative headache characteristics were significantly associated with surgical outcomes: unilateral headache was independently associated with higher odds of seizure-free within 1 year after surgery, while a higher preoperative headache VAS score was independently associated with lower odds of seizure-free within 1 year after surgery. These results suggest that comorbid headache itself does not directly affect short-term surgical outcomes in DRFE patients, but unilateral headache and lower preoperative headache intensity may serve as potential clinical indicators for the favorable short-term surgical outcomes in DRFE patients with comorbid headache. In clinical preoperative evaluation, detailed collection of headache characteristics should be emphasized to more accurately guide individualized treatment decisions.

## Data Availability

The original contributions presented in the study are included in the article/supplementary material, further inquiries can be directed to the corresponding authors.
